# Implementation of a pilot community-based psychosocial intervention for patients with psychoses in Chile and Brazil: a comparative analysis of users' perspectives

**DOI:** 10.1017/gmh.2021.10

**Published:** 2021-04-27

**Authors:** Saloni Dev, Tanvi Kankan, Drew Blasco, PhuongThao D. Le, Martin Agrest, Gabriella Dishy, Franco Mascayano, Sara Schilling, María José Jorquera, Catarina Dahl, Maria Tavares Cavalcanti, LeShawndra Price, Sarah Conover, Lawrence H. Yang, Rubén Alvarado, Ezra S. Susser

**Affiliations:** 1 Teachers College, Columbia University, New York, NY, USA; 2 School of Global Public Health, New York University, New York, NY, USA; 3 Proyecto Suma, Buenos Aires, Argentina; 4 Mailman School of Public Health, Columbia University, New York, NY, USA; 5 Division of Behavioral Health Services and Policy Research, New York State Psychiatric Institute, New York, NY, USA; 6 Escuela de Salud Pública, Facultad de Medicina, School of Public Health, Universidad de Chile, Santiago, Chile; 7 Primary Care and Family Health Department, Universidad de Chile, Santiago, Chile; 8 Pan American Health Organization/World Health Organization, PAHO/WHO Office in Brazil, Brasília, Brazil; 9 Institute of Psychiatry, Federal University of Rio de Janeiro, Rio de Janeiro, Brazil; 10 National Institute of Mental Health (NIMH) and National Institutes of Health, Washington, DC, USA; 11 National Heart, Lung, and Blood Institute, National Institutes of Health, Bethesda, MD, USA; 12 Center for the Advancement of Critical Time Intervention, Silberman School of Social Work, Hunter College, City University of New York, New York, NY, USA; 13 New York State Psychiatric Institute, New York, NY, USA

**Keywords:** Community-based mental health services, critical time intervention, Latin America, psychosis, qualitative methods, task-shifting

## Abstract

**Background:**

Few studies provide clear rationale for and the reception of adaptations of evidence-based interventions. To address this gap, we describe the context-dependent adaptations in critical time intervention-task shifting (CTI-TS), a manualized recovery program for individuals with psychosis in Rio de Janeiro, Brazil and Santiago, Chile. Implications of the adaptations – incorporating a task-shifting approach and modifying the mode of community-based service delivery – are examined from users' perspectives.

**Methods:**

A secondary analysis of in-depth interviews with CTI-TS users (*n* = 9 in Brazil; *n* = 15 in Chile) was conducted. Using the framework method, we thematically compared how participants from each site perceived the main adapted components of CTI-TS.

**Results:**

Users of both sites appreciated the task-shifting worker pair to provide personalized, flexible, and relatable support. They wanted CTI-TS to be longer and experienced difficulty maintaining intervention benefits in the long-term. In Chile, stigma and a perceived professional hierarchy toward the task-shifting providers were more profound than in Brazil. Engagement with community-based services delivery in homes and neighborhoods (Chile), and at community mental health centers (Brazil) were influenced by various personal, familial, financial, and social factors. Uniquely, community violence was a significant barrier to engagement in Brazil.

**Conclusion:**

CTI-TS’ major adaptations were informed by the distinct mental health systems and social context of Santiago and Rio. Evaluation of user experiences with these adaptations provides insights into implementing and scaling-up task-shifting and community-oriented interventions in the region through the creation of specialized roles for the worker pair, targeting sustained intervention effects, and addressing socio-cultural barriers.

## Introduction

Recently there have been numerous efforts to adapt evidence-based interventions (EBIs), typically developed in high-income countries (HICs), to the contexts of low- and middle-income countries (LMICs; Escoffery *et al*., 2018; Tiley and Kyriakopoulos, 2018). Adaptation involves calibration of the EBI to fit a different setting through careful consideration of the setting's health system, cultural norms and values, and available resources, while also maintaining an optimal degree of fidelity to its core components. Although these practices are commonplace globally, few studies have clearly detailed the rationale for such adaptations in the context of the existing local initiatives and evaluated their implementation outcomes (Escoffery *et al*., 2018; Alarcón *et al*., 2020). This paper seeks to describe several context-dependent adaptations of a manualized recovery program for individuals with psychosis in two major Latin American (LA) cities, and examine their implications from the perspectives of users who participated in the program.

## Critical time intervention (CTI): an EBI

CTI is a structured manualized EBI originally developed to improve continuity of care for those with severe mental illness transitioning from homeless shelters to community living in New York city (Susser *et al*., 1997). CTI has since been successfully adapted and implemented to address the needs of other vulnerable populations (e.g. veterans and prisoners; Herman *et al*., 2007; Kasprow and Rosenheck, 2007; Dixon *et al*., 2009). CTI's core components include: (a) time-limited duration (~9 months); (b) community-based services; and (c) three structured chronological phases: (1) initiating family and community support networks, (2) monitoring strength of support networks, and (3) gradual termination (Herman and Mandiberg, 2010; Center for the Advancement of CTI, 2014).

## Adapting CTI for the LA setting

In 2014–2015, a pilot randomized control trial of an adapted version of CTI was conducted to promote community reintegration and recovery among individuals with a range of affective and non-affective psychosis in two LA settings: Rio de Janeiro, Brazil, and Santiago, Chile. Brazil and Chile have both made considerable progress in their psychiatric reform to promote social integration within mental health services (Mateus *et al*., 2008; Alvarado *et al*., 2012). However, they have distinct implementation settings, as shown in Table 1.

**Table 1.** Comparison of implementation settings in Chile and Brazil

	Chile	Brazil
Population	17.77 million [World Health Organization (WHO), 2014*b*]	202.03 million (WHO, 2014*a*)
Gross domestic product per capita (purchasing power parity) in 2018	$15 923	$8921
Mental health policies & legislation	No mental health law	Mental Health Law (Federal Government of Brazil, 2001), based on the Caracas Declaration and in accordance with WHO guidelines, which guarantees civil rights for people with mental illnesses (Mateus *et al*., 2008)
	Coverage for certain physical/mental health conditions through specific programs	All mental health conditions covered but with severe budgetary restrictions
Total number of mental health professionals per 100 000 population	16.3 (not specified if only public sector) (WHO, 2014*b*)	30.8 (public sector only) (WHO, 2014*a*)
Psychiatric beds & annual inpatient admissions per 100 000 population	6.5 beds (27.8 annual admissions) (WHO, 2014*b*)	11.6 beds (216.3 annual admissions) (WHO, 2014*a*)
Allocation of funding to mental health services	2.16% of total health budget (data from 2014) (Ministerio de Salud de Chile, 2017)	2.3% of total health budget (Trapé & Campos, 2017)
Treatment gap	**61.5%** for any disorder; **39.8%** for severe disorders (Kohn *et al*., 2018)	**78.1%** for any disorder; **67.2%** for severe disorders (Kohn *et al*., 2018)
Community mental health strategies	1. Integrate mental health care into primary care 2. Develop local community mental health centers	Develop community-based care programs as alternative to asylums
Community mental health centers (CMHCs)	*Centro Comunitario de Salud Mental* (COSAMs) and *Centro de Salud Mental* (CESAMs)	*Centro de Atenção Psicossocial* (CAPS)
Services offered by CMHCs	A variety of pharmacological and psychosocial interventions offered by teams of specialized professionals, but community workers and home visits less common	A variety of pharmacological and psychosocial interventions offered by teams of specialized professionals, including some home visits and provision of additional community resources; these are restricted due to lack of specialized community worker/case manager role
Socio-economic characteristics of population/region served	Predominantly low income and also middle-low income community residents	Predominantly low income community residents
	Territory marked by moderate violence and precarious living situations	Territory marked by armed conflicts due to narcotraffic, violence, and poverty

After careful consideration of the sites' contexts, two major adaptations were enacted. First, given the limited number of mental health professionals in LA, a task-shifting model was incorporated, hence the adapted intervention was named critical time intervention-task shifting (CTI-TS). Second, due to differences in the structure of Brazil and Chile's community mental health systems (Table 1), and concerns around violence in the community settings in Rio, the intervention in Brazil primarily took place in the community mental health centers (CMHCs; i.e. *Centro de Atenção Psicossocial*-CAPS), while in Santiago it was conducted within users' homes and surrounding neighborhoods. These adaptations were drafted collaboratively by researchers at Columbia University, Universidade Federal do Rio de Janeiro, and Universidad de Chile, and informed by their experience developing and piloting CTI in Brazil (Mascayano *et al*., 2019).

## Adapting to the mental health system through task shifting

While CTI is usually delivered by health-related professionals (e.g. social workers, case managers, and mental health counselors), CTI-TS’ intervention delivery was shifted to a synergistic worker pair: a community mental health worker (CMHW) and a peer support worker (PSW). The CMHWs were individuals committed to providing mental health services and social support within community settings, while the PSWs could share their own mental health recovery experiences with the CTI-TS users, in line with the emphasis placed by LA culture on solidarity and social support (Sanabria, 2007). This ‘worker pair’ were trained and supervised to collaborate with users to identify barriers to their recovery and develop a sustainable plan to engage in and use community supports and resources during and after CTI-TS (Conover and Restrepo-Toro, 2013). They also used their knowledge to increase users' autonomy, strengthen their linkage to health services, and expand their support networks.

### Implementation of the task-shifting adaptation

Given the differences in each site's mental health systems (Table 1), the implementation of the task-shifting adaptation, along with the selection of the worker pair, varied between sites.

In Santiago, task-shifting was incorporated in a traditional sense whereby the worker pair functioned independently from mental health professionals to carry out their specific tasks. Additionally, the Chilean CMHCs – i.e., *Centro Comunitario de Salud Mental* (COSAMs) and *Centro de Salud Mental (*CESAMs) – had limited experience providing recovery-oriented services outside the centers. Therefore, the worker-pair in Santiago held a special role of supporting users in their recovery, and engaging their families and other community members in their recovery process. PSWs were selected from the participating CMHCs to capitalize on established relationships with professionals and familiarity with the community. Those experienced in working with vulnerable populations but with no formal training in the implementation of mental health services were selected to be CMHWs.

In Rio, however, a more collaborative version of task-shifting, i.e. ‘task-sharing’, was adopted. In task-sharing, work is not entirely shifted from one level of providers to another but is shared amongst the two groups. This ‘allows a limited number of specialists to practice in teams with other providers and community resources to reach populations in need’ (Hoeft *et al*., 2018) in a more supervisory, consultant role. Community work was already part of CAPS providers' responsibilities but delivery was limited due to the scarcity of personnel and resources. Thus, the Rio worker pairs collaborated closely with the mental health professionals to deliver services. PSW appointments were made from outside of the participating CMHCs, based on the recommendation of users' groups. CMHWs were selected from among graduates of a community health worker training course who had experience working with people with severe mental illnesses.

In both sites, given the limited availability of PSWs and CMHWs, they were matched to work as a pair based on the judgement of the implementation team around compatibility.

## Adapting to the community conditions through modifying the intervention setting

CTI-TS aimed to retain the original community-based intervention delivery aspect given the cultural value ascribed to family and societal bonds (Sanabria, 2007), thereby empowering users to engage with their communities and supporting them in establishing long-term support networks. Further, the intervention setting was modified in each city to ensure its fit with the local mental health infrastructure and community conditions.

### Implementation of community-based component

In LA, there is a strong and growing network of CMHCs, which are a part of the community-based approach to address social dimensions of health in accordance with the Caracas Declaration in 1990 (Organización Panamericana de la Salud/Organización Mundial de la Salud, 1990; Vasquez *et al*., 2019). However, important differences between the community mental health systems of Brazil and Chile that were identified by the local implementation teams further informed the adaptation and implementation of CTI-TS (Table 1). Particularly, given the mental health reform in Brazil, the network of CAPS was very experienced in providing mental health care within communities through psychosocial interventions, and recovery promoting programs via economic empowerment and social reintegration (Campos *et al*., 2009; Kantorski *et al*., 2009). Hence, and amidst the significant community violence in Rio, it was deemed suitable to capitalize on CAPS’ experience and integrate PSWs and CMHWs within its infrastructure. In contrast, although Chilean COSAMs and CESAMs have attempted to move towards a community-based model of mental health care, the professionals continued to provide services predominantly within these health facilities rather than in community-settings (WHO and Ministerio de Salud, 2007; Mascayano and Montenegro, 2017). In light of this, the worker pairs in Santiago delivered services in the local communities, in and nearby users' homes as initially intended, to get to know and support the users, their families, and their needs, in order to complement the services that were already provided by the COSAMs and CESAMs.

Table 2 summarizes the adaptations and their differential implementation of CTI-TS at the two sites. These features reflect a deliberate attempt to maximize the contextual fit of CTI-TS, according to the aforementioned mental health systems and social context (prevalence of community violence) of Santiago and Rio. Nevertheless, the core elements of the intervention – i.e. the task-shifting/task-sharing strategy and the community-based aspect – remained the same due to the dearth of mental health professionals as well as the cultural profile of both the settings. Other elements of the intervention were also similar across sites; for example, the structure and contents of the worker-pair trainings were standardized and manualized (Mascayano *et al*., 2019).

**Table 2.** Summary of features of CTI-TS implementation

	Santiago, Chile	Rio de Janeiro, Brazil
CTI-TS sites	Five COSAM or CESAM sites in three catchment areas	Three CAPS sites in single catchment area
Implementation of task-shifting adaptation	*Task-shifting*: Worker pairs functioned independently from (though in communication with) the mental health professionals to carry out their specific tasks	*Task-sharing*: Worker pair worked more closely with CMHC mental health professionals, who functioned as supervisors. This allowed CMHC professional involvement without imposing as significant demands on their time as would have been required if they carried out the tasks alone
Intervention delivery setting	In user's homes and neighborhoods, and local organizations	Primarily in CAPS, with some community visits

As neither settings had previously tested the integration of the task-shifting and community-based components into their mental health systems, there is currently scarce evidence to understand how these adaptations are experienced by various stakeholders including mental health service users. This paper analyzes users' perspectives and experiences regarding the implementation of these adaptations in Brazil and Chile. Exploring users' perceptions and experiences contributes valuable stakeholder perspectives on the mental health system- and culture-related challenges and potential innovations in scaling up this and other task-shifting, community-based interventions in Latin America.

## Methods

### Data collection

This analysis draws from data collected as part of a larger study that evaluated the pilot CTI-TS study, whereby qualitative interviews were conducted among various stakeholders (users, providers, and CMHC administrators). These interviews aimed to evaluate potential barriers and facilitators to the implementation and scale-up of the piloted task-sharing strategy and community-based delivery. The current, secondary analysis utilizes the semi-structured interviews among CTI-TS users from the two sites (*n* = 9 in Brazil; *n* = 15 in Chile)^‡^^1^. Table 3 summarizes the socio-demographic characteristics of the sample.

**Table 3.** User socio-demographic variables

Characteristic	Chile (*n* = 15) (%)	Brazil (*n* = 9) (%)	Across sites (*n* = 24) (%)
Gender			
Male	10 (66.67)	4 (44.44)	14 (58.33)
Female	5 (33.33)	5 (55.56)	10 (41.67)
Age range			
21–29	2 (13.33)	3 (33.33)	5 (20.83)
30–39	6 (40.00)	4 (44.44)	10 (41.67)
40–49	5 (33.33)	1 (11.11)	6 (25.00)
50–65	2 (13.33)	1 (11.11)	3 (12.50)
Psychosis diagnosis^a^			
Affective	8 (53.33)	3 (33.33)	11 (45.83)
Non-affective	7 (46.67)	6 (66.67)	13 (54.17)
Education level			
No school/incomplete or complete grade school	5 (33.33)	2 (22.22)	7 (29.17)
Some high school	2 (13.33)	1 (11.11)	3 (12.50)
High school graduate	3 (20.00)	6 (66.67)	9 (37.50)
More than high school	5 (33.33)	0 (0.00)	5 (20.83)
Employment status			
Employed (regular)	3 (20.00)	1 (11.11)	4 (16.67)
Occasional or unstable work	5 (33.33)	0 (0.00)	5 (20.83)
Housewife/housework	1 (6.67)	2 (22.22)	3 (12.50)
Retired/Pensioner	4 (26.67)	3 (33.33)	7 (29.17)
Unemployed/other	2 (13.33)	3 (33.33)	5 (20.83)
Living situation			
Living alone	3 (20.00)	1 (11.11)	4 (16.67)
Living with spouse/partner (w/ or w/o children)	4 (26.67)	2 (22.22)	6 (25.00)
Living with parents or other relatives	7 (46.67)	5 (55.56)	12 (50.00)
Other living situation	1 (6.67)	1 (11.11)	2 (8.33)
Income source			
Earned income/salary	6 (40.00)	1 (11.11)	7 (29.17)
Pension/government assistance	4 (26.67)	5 (55.56)	9 (37.50)
No income	3 (20.00)	1 (11.11)	4 (16.67)
Other (family aid, unstable income, rent, etc.)	2 (13.33)	2 (22.22)	4 (16.67)

a Affective psychosis is psychosis (e.g. delusions or hallucinations) occurring only in the context of a mood disorder. Non-affective psychosis includes other disorders of psychosis; including schizophrenia, brief psychotic disorder, etc. Those with psychosis due to organic conditions were excluded.

### Data analysis

The interviews were conducted by trained psychologists in each site's respective local language (Brazil: Portuguese; Chile: Spanish) and lasted between 45 and 60 min. Audio recorded interviews were transcribed verbatim in the original language and were then translated to English by native bilingual research personnel.

Data were analyzed using the framework approach wherein the research team: (1) gained familiarity with the data by coding the first few transcripts; (2) inductively developed a thematic framework; (3) indexed codes according to the framework and resolved disagreements through consensus; (4) charted how themes and codes are connected to each other; and (5) mapping how the themes were similar or different across sites, and interpreted the results (Srivastava and Thomson, 2009). We developed a coding framework based on the core components of CTI-TS and prior analyses of the qualitative evaluation data (Agrest *et al*., 2019). True to the framework method, open coding was also used, to allow for identification of additional themes. To ensure accuracy of data interpretation, reflexive memos were written throughout the coding process and local researchers from each site verified whether interpretations were consistent with observations on the ground during the implementation of the CTI-TS.

### Ethical review

This study was approved by the Columbia University, Brazil's National Commission for Ethics in Research (*Comissão Nacional de Ética em Pesquisa*), and the Universidad de Chile. Informed consent was obtained from all eligible participants prior to conducting the interviews.

## Results

Our findings compare participants' perspectives in Santiago and Rio on the two adapted components of CTI-TS described above.

### Task shifting adaptation

#### Similarities across Santiago and Rio

##### Desire for CTI-TS to be longer

While they had been told that CTI-TS was time-limited (9 months), participants wished the intervention lasted longer: Santiago Participant 6 (P6) elaborated:

‘I would like them to continue to come to see me … I wanted to talk more *…* say something about myself … they said they had to go and I was left with the wish.’

Rio P2 suggested the length for the intervention *‘should be 12 months, 13 months’* for her family to properly engage with the worker-pair.

##### Difficulties sustaining intervention benefits

Similarly, several participants across sites highlighted that CTI-TS benefits were difficult to sustain without the worker pairs' support after intervention termination. One participant (Santiago P12) described how his family interactions had been different under the worker pair's influence: *‘Yes,* [*my family*] *changed, they were more like ‘silk’ (softer). Now they are ‘heavier’ (moody) … because I buy cigarettes, ask for money.’* Another (Rio P1) expressed he no longer engaged in the activities as he had with the worker pair when they used to visit.

##### Worker pair flexibility and reliability

Participants in both sites appreciated the worker pairs' flexibility in scheduling meetings, which was often contrasted with that of local mental health services, especially in Santiago. For example, Santiago P5 described how CTI-TS workers adjusted to his timing and location requirements, whereas sudden appointment cancellations often occurred when coordinating with mental health professionals. He also described their reliability and punctuality:

“They called every time they were coming “we are on our way”, “we'll be there in half an hour, is that ok?” *…* That was really important, because … most clinics … give you an appointment and they see you very late, and one has to wait. That didn't happen [with the CTI-TS workers].”

Rio P6 noted how the worker pair would wait for him at the center since he had no phone he could be reached on: *‘They knew the day I used to come and then they came too.*’

##### Worker pairs provide relatable, personalized support

Worker pairs' competency in meeting the specific needs of participants was generally appreciated across both sites. Participants acknowledged the motivational, emotional, and instrumental support they provided and the benefits it led to including continued adherence to the intervention and increased user mobility that facilitated community integration.

A few participants who did not have supportive families especially appreciated receiving support from the worker pair:

‘*…* they feel alone [*…*] So to have someone who isn't from the family … That's what happened to me … there were people who weren't from my family who were worried about me and were with me.’ (Santiago P3)

Participants across sites also reflected on how their shared experiences with the PSWs fostered their relationship with them through feelings of mutuality and empathy.

‘When I heard she had a problem, I became even more interested. Because then, I thought, ‘Well, she has the same problem as me, she'll understand a little bit about the situation.’’ (Rio P7)

Participants identified with the worker pair more often than professional mental health workers, apparently because their shared socioeconomic backgrounds and lived experiences facilitated easier communication. Rio P4 described, ‘*I liked* [*the worker pair*] *more* [*than the professionals*] *… because they speak my language … They're more open … I felt more comfortable.’*

Participants across sites expressed that the care provided by the worker pair complemented that of the professionals and the support they extended was more personalized than the care delivered through the CMHC:

‘It seemed that [the CTI worker] was more interested in the things that I was talking about *…* [the CAPS professionals] are dealing with several people.’ (Rio P5)

#### Context-specific findings

##### Preference for professionals' role

While most participants across sites felt that the worker pair were adequately trained for their respective roles, and that these roles were not supposed to replace that of the professionals, some were doubtful and expressed more trust in a professional's competence as a result of their greater education and experience. This perceived professional hierarchy was particularly pronounced in Santiago, as illustrated by Santiago P7:

‘I felt that the [CMHW] … needed *…* more … work with your concepts … like she doesn't understand *…*. She doesn't get much from what we're talking [about]’

Another participant stated, ‘*Since* [*the worker pair*] *aren't doctors, they are normal people … they don't have so many resources to help me.*’ (Santiago P8)

##### Stigma against worker pairs and misunderstanding of worker roles

Santiago P8 regarded the worker pair as ‘*mentally ill*’ and hence not competent, indicating a lack of differentiation of the two worker roles, and a degree of internalized stigma. Santiago P5 indicated a different misinterpretation of the roles: *‘The peer was the assistant of the* [*CMHW*] *… Because* [*the PSW*] *is a patient … He is not a professional … only a patient.’* Such role confusion was not common among Rio participants.

Stigma toward the PSW from participants, family and/or community was observed in Santiago, but not in Rio. Santiago P10 noted that his aunts made prejudiced comments about the PSW after they briefly met him: *‘One can see he has schizophrenia, one can see that it's a guy with problems.’*

##### Intervention timing

Finally, participants in Santiago expressed far more positive remarks about how the intervention ‘*came just in the time*’ for people who needed the help (Santiago P5) compared to their Rio counterparts. While Rio participants approved of the timing, they did not appear to give it as much importance.

### Community-based component: adaptation to participant's home in Santiago *v*. mental health center in Rio

#### Similarities across Santiago and Rio

##### Financial barriers

When asked if she ever thought of giving up the intervention, Rio P2 noted, ‘*It was more for this financial reason, wasn't it?’* Participants in both Santiago and Rio expressed that personal financial limitations interfered with intervention engagement by making it difficult to travel for activities suggested by the worker pair (or in the case of Rio, to visit the CAPS), or to communicate with the workers in the absence of money to purchase a phone. Santiago P15 stated: ‘*Sometimes I didn't have money to go* [*to the CMHC*] *… they would write me a note so they didn't charge me*.’

##### Personal and familial factors influencing intervention engagement

Physical illness and impairment, medical side effects, symptoms, low motivation, and family protectiveness apparently limited users' engagement in community activities. Rio P5 explained: *‘I might even go, but my mom thinks that I'm going to do the same thing I did when I got into the crisis.’* Meanwhile, family support, self-motivation toward recovery, and a positive help-seeking attitude were viewed as facilitating intervention engagement; Santiago P3 described how situational stressors, such as a house fire or her newly living alone, did not interfere with her CTI-TS engagement because her family helped her with her other responsibilities.

##### Influence of social benefits and difficulty obtaining them

Social policies in both countries enhanced the implementation of CTI-TS by encouraging mobility and financial flexibility. Specifically, guaranteed coverage for prioritized medical conditions including schizophrenia helped reduce costs for participants in Santiago, and disability-related benefits for transportation in Rio served CTI-TS users and PSWs. As Rio P8 explained, ‘[*The PSW*] *had a pass card that gave access to two people … whenever I went out with him, as much as I was out of money, I did not pay for the ticket because he used his card…’* In both cities, administrative bureaucracy could apparently interfere with the attainment of such benefits. Rio P2 cited stringent criteria to receive benefits and difficulties around technology that had to be used to access them. Santiago P13 reflected that this made him anxious, with him feeling that rather than giving *‘… them every detail of what you do, what you buy, what you earn,’* he needed to ‘*be in the streets, looking for money … working.’*

#### Context-specific findings

##### Community violence affects meetings

Rio participant interviews confirmed that community violence was a significant barrier for community-based mental health care, even when the intervention was adapted to take place primarily at the CAPS, away from the insecurity of the participants' neighborhoods: *‘… there is a great insecurity, you know? Every day has …* [*a*] *… shootout.’* (Rio P9) The violence negatively affected users' participation in the CTI-TS activities and led to multiple appointment cancellations. Rio P3 commented that while residents ‘*get used to*’ the violence and gunfire, at that time ‘*there is no way to get out.*’.

A few Santiago participants acknowledged the occurrence of community violence (Santiago P15: ‘*there are people who sell drugs who fight with knives, who fight with guns*’) but it did not affect their movement around their immediate neighborhood, or CTI-TS meetings.

##### Comfort of meeting at home *v*. stigma-based fears at CMHC

Santiago's home-based meetings appeared to greatly facilitate intervention engagement; two participants explicitly mentioned that their home was ‘like a refuge,’. Another Santiago participant's (P8) stigma toward others with mental illness did not interfere with the intervention since it was based entirely in his home and neighborhood. In Rio, stigma negatively influenced some participants' motivation to attend meetings at CAPS due to its association with hospitalization and people with severe levels of mental illness.

‘[At the previous clinic] *…* it is *…* different, they separated the people … who were not so well *…* And [at CAPS] it's all together … a lot of people in the same place.’ (Rio P3)

##### Ease of home visits *v*. issues with travel to CMHC for CTI

Santiago participants acknowledged the convenience of the workers visiting them at home; the same was true in Rio when such home visits were made. However, Rio participants' ability to attend meetings at CAPS was sometimes affected by dependence on family members for transportation, or financial issues. Rio P4 said, ‘*I think that the day I couldn't come, it was because* [*my daughter*] *would not let me come* [*alone*] *… She was afraid.’*

##### CTI workers' outsider perspective on participant's interaction with local MH services *v*. close partnership with CMHC staff

The physical independence of CTI-TS from CMHC in Santiago allowed the worker pair to have an outsider perspective on the participants' experience with local mental health services; they could observe each participant's particular context and help them navigate the CMHC or hospital system to obtain treatment.

‘[The CTI-TS workers] kept following up with me during the process … It's necessary that they see; that they evaluate from the outside how one as a user perceives the [mental healthcare] system’ (Santiago P7)

Conversely, in Rio the CTI-TS workers worked closely with the CAPS provider team. Therefore, participants were able to comment on the integration (or lack thereof) of the worker pair and the CAPS staff. Indeed, at times Rio participants appeared to find it difficult to distinguish between CAPS and CTI-TS staff.

##### Home visits allow unique insights into participant's status and needs

Santiago participants highlighted how the home visits enabled the worker pair to not only gain insight into their daily and social life, but also their motivation and attitude toward treatment. As Santiago P13 stated: ‘*You can see the person's circumstances … the way he lives … if he wants to move on, if he is really willing … if he really needs help*.’ This participant felt the worker pair's community visits provided screening and safeguard for participants vulnerable to high-risk behavior, and could help identify those needing governmental benefits and care:

‘[The worker pair could] *…* know better about the situation of each person and provide the necessary help for that person not to fall into a risk *…* A greater risk that may lead to suicide, to going crazy … becoming a drug consumer.’ (Santiago P13)

## Discussion

We described the two major contextual adaptations made to the CTI model, namely the addition of a task-shifting component and a change in the setting of the intervention, in light of the social characteristics and mental health system of two LA cities, Santiago, Chile, and Rio de Janeiro, Brazil. Participants spoke to both positive and negative implications of the contextual adaptions described regarding the CTI-TS model. Additionally, the observed differences and similarities – identified from the users' interviews – across the two sites may have important implications for further adaptation and scale-up of CTI-TS, as well as other task-shifting based, community-oriented interventions in the region.

### The worker pair: a promising addition to the community-based, recovery-oriented mental health services

Users widely acknowledged the reliability, scheduling flexibility, and more personalized support from the worker pair as compared to the mental health professionals. A history of CMHWs in both countries likely contributed to participants' acceptance and comfortability with CTI-TS’ task-shifting model. Chile has involved ‘community health agents’ (*agentes comunitarios de salud*) and ‘community mental health monitors’ (*monitores de salud*) for decades in rolling out its community mental health approach (Marconi, 1971; Encina and Minoletti, 2016; Ministerio de Salud, 2016). In Brazil, the community health agents (*agentes comunitários de saúde)* date back to 1980, and the CAPS have since incorporated and implemented many community-based interventions. Consequently, there may be great potential for the creation of specialized roles for CMHWs and PSWs, along with the appropriate regulatory framework, to provide recovery-based mental health services in LA (Agyapong *et al*., 2016; Daniels *et al*., 2017). However, as identified in Santiago, preference for mental health professionals due to their relatively higher education and training, as well as stigma against the PSW are potential areas of concern that would need to be further investigated to ensure the success of these specialized roles.

### Sustaining the positive impact of time-limited, task-shifting-based intervention

Users' comments regarding CTI-TS timing may reflect differences in the two countries' mental health system features. Given the long existence of CAPS in Rio, and since the initiation of CTI-TS might have blended into their ongoing treatment, participants did not remark as much on its timing. In Santiago, however, CTI-TS filled a void given the relative lack of community-based services, where participants could clearly experience the introduction of new services; hence, its critical timing was often appreciated by users. Relatedly, in both locations, some participants expressed that the positive effects were not sustained. Future research should explore strategies to ensure continued post-intervention support, such as potentially extending the intervention's duration when implemented by task-shifting providers, in order to better sustain the goals of CTI-TS in the long-term.

### Addressing socio-cultural barriers: violence and mental health stigma

The community violence interfering with the CTI-TS implementation in Rio was a significant social barrier at the site. This was anticipated and hence, the intervention was grounded within CAPS. However, users and CTI workers experienced significant limitations traveling back and forth from home to CAPS, especially when they were located in particularly violent neighborhoods. Despite users' abilities to ‘normalize’ and adapt to violence in their communities, taking part in activities in other community settings (e.g. CAPS) or receiving outsiders (e.g. CTI workers) represented a tough hurdle not completely overcome by the intervention.

Users' responses to the adaptations also revealed important connections to LA's broader socio-cultural context. Stigma toward others with mental illness was prevalent in both settings and posed challenges to intervention implementation in different ways. Though it was hypothesized that participants would prefer interacting with lay providers from a similar socioeconomic and mental health background, some Santiago users and families expressed stigmatizing attitudes towards the PSWs, viewing them as less competent and as ‘assistants’ to CMHWs. This stigma may be propelled by the prevalent social hierarchies in Chile whereby identification with and acceptance by those with a higher social standing (such as the doctors or mental health professionals) is valued (Bochniak-Piasecka, 2013). Similarly, in Rio, some users' stigma toward other mental health service users made them reluctant to go to CAPS for the intervention. Accordingly, implementation and dissemination of community-based EBIs for individuals with psychosis may benefit from including components that address the stigma surrounding severe mental illness (Agrest *et al*., 2019). Our results are consistent with the broader mental health and stigma literature that interventions involving peers should also directly target internalized stigma among service users (Firmin *et al*., 2017; Pyle *et al*., 2018; Jones *et al*., 2019). The findings further extend support for instituting specialized roles for the worker pair that may help in navigating the social hierarchies and enhancing acceptability among users.

### Enhancing community-based services delivery

While participants often spoke to the positive aspects of CTI-TS’ community-based delivery, negative implications were also noted including stigma-based fears of receiving the intervention primarily at CAPS in Brazil. While our results lend support to the notion that CTI-TS can be adapted to fit local conditions, future research should examine how to enhance CMHC-based delivery through exploring stigma-related barriers as well as through understanding the training and time needed for the worker pair to integrate into the CMHC. Users in both settings also noted that this community-based element was affected by matters such as finances, access to social benefits, physical symptoms, motivation level, family support, and neighborhood conditions. These challenges necessitate that researchers and stakeholders advocate for adequate public funding to remove financial barriers for users, and for including comorbidities in primary diagnostic categories to alleviate physical symptoms that may act as barriers to receiving interventions (Mechanic, 2002). Further, given the value placed by LA culture on family, and the social and emotional support they provide users, future implementation and scale-up efforts could incorporate additional methods to engage users' family members, as enhanced family support may facilitate the sustainment of benefits post-intervention (Robila, 2016). Additionally, studying neighborhood conditions that potentially hinder users from accessing mental health services within their communities or at CMHCs may enhance engagement. In fact, the distinct delivery settings in Brazil and Chile in our study might explain some of the differences in user experiences observed above such as with neighborhood violence and social stigma. When culturally and logistically appropriate, services delivery within users' homes may facilitate better treatment engagement through convenience and insights into users' needs, further highlighting the importance of the posited specialized roles for the worker pair.

### Limitations and strengths

This analysis should be considered in light of several limitations. First, the thoroughness of experiences narrated by the users may have been impacted by their diagnosis. Nonetheless, being symptomatic during interviews did not prevent the users from offering interesting inputs about their experiences with CTI-TS. Second, due to relatively small sample size, these results may not be representative of all users' perspectives. The cultural nuances presented here are based on endorsements made by the users, but we anticipate more sociocultural influences at play that would need to be explored further. Future analyses should seek to utilize a larger and more diverse sample, including perspectives from other stakeholders. Third, this is a secondary analysis of data that was not originally intended to answer our research question. However, the original focus on identifying barriers and facilitators to the scale-up of CTI-TS lended itself to the current analysis. Fourth, the qualitative approach adopted in the analysis of our data has excluded clinical and other quantitative measures that were beyond the scope of this study. The limited number of participants additionally impedes differential intergroup analysis (e.g. by diagnosis or other relevant clinical considerations). Lastly, because this intervention model is new to the LA contexts and that few service users have experienced the type of care that were provided through the CTI-TS, much of their perceptions, positive or negative, might be due to the novelty of the intervention, and not specific to the highlighted adaptations.

The study has uniquely contributed to the literature by detailing the nuances of users' reactions, both positive and negative, to the contextual adaptations from the two sites. An additional strength is its detailed rationale for adaptations, which may allow future researchers and professionals to better adapt CTI and other EBIs. Additionally, evaluating the users' receptions of the adaptations from a qualitative perspective may contribute insight on the challenges of maintaining the integrity of the core intervention components and fidelity to the intervention model.

## Conclusion

Bridging a significant gap in the literature, this paper identified and detailed the rationales for adaptations made in the pilot trial of an evidence-based psychosocial intervention to assist the recovery of individuals with psychosis in two major cities in LA. Considerations of the distinct mental health system and social characteristics of the two settings resulted in the adoption of the task-shifting approach, and different modalities of community-based service delivery. Evaluation of user experiences with these adaptations illustrated the promising position task-shifting- and community-based mental health interventions hold in LA. Further, it highlighted the opportunities and challenges posed by socio-cultural influences (e.g. community violence, family engagement and mental health stigma) in the implementation of the adaptations. Implementation and scale-up of interventions such as CTI-TS may greatly benefit from addressing the aforementioned barriers and from further research on strategies to sustain positive intervention effect, combat stigma against PSWs, and navigate social hierarchies.

## Footnotes

‡ The notes appear after the main text.

1 The pilot CTI-TS study included a sample size of 30 users in Santiago and 25 in Rio in the treatment arm.
